# Supplementary material for: EnzML: multi-label prediction of enzyme classes using InterPro signatures
Source: BMC Bioinformatics. 2012 Apr 25;13:61. doi: 10.1186/1471-2105-13-61 (PMC3483700; doi:10.1186/1471-2105-13-61)
Supplement: Addtional file 5 — The Java code to format the data files, evaluate and predict. The file enzml_java_code.tar.gz contains the Java code used to format database data to ARFF and XML formats, to execute cross and train-test (jackknife) evaluations and to record evaluation results to database. More information is included in the readme.txt file and the Javadoc files. The code can be used with a MySQL database. To use a different database software, other JDBC drivers might be required. [file 1471-2105-13-61-S5.gz › java_code/enzml2011/doc/uk/ac/ed/inf/enzml/weka/ArffProperties.html]

ArffProperties


---


|  |  |  |
| --- | --- | --- |
| **Overview** \| **Package** \| **Class** \| **Use** \| **Tree** \| **Deprecated** \| **Index** \| **Help** | |  |
| **PREV CLASS**   **NEXT CLASS** | **FRAMES**    **NO FRAMES**     **All Classes** |  |
| SUMMARY: NESTED \| FIELD \| CONSTR \| METHOD | DETAIL: FIELD \| CONSTR \| METHOD |  |


---


## uk.ac.ed.inf.enzml.weka Class ArffProperties

```
java.lang.Object
  uk.ac.ed.inf.utils.Initialised
      uk.ac.ed.inf.enzml.weka.ArffProperties
```

**All Implemented Interfaces:**: uk.ac.ed.inf.utils.Initialisable

**Direct Known Subclasses:**: MulanArffProperties

---

``` public class ArffProperties extends uk.ac.ed.inf.utils.Initialised ```

Contains the properties for a Weka ARFF file
It contains: 1. the properties read from properties file 2. the path of the
properties file itself 3. the name of the arff file including a timestamp 4.
(optional) some statistics for the data (number of attributes, classes,
instances.

**Version:**
:   24-Mar-2007

**Author:**
:   Luna De Ferrari luna.deferrari@ed.ac.uk

---

| **Field Summary** | |
| --- | --- |
| `static java.lang.String` | `ARFF_FILE_PATH_PROP`             the file path where the ARFF file will be written, inclusive of file name. |
| `static java.lang.String` | `CLASS_NAME_PROP`             The class name. |
| `static java.lang.String` | `DATA_PROPERTIES_FILE_PATH_PROP`             the path to the properties file itself, from which the other properties were read |
| `static java.lang.String` | `DATASET_NAME_PROP`             the experiment short name |
| `static java.lang.String` | `DESCRIPTION_PROP` |
| `static java.lang.String` | `INSTANCE_ATTRIBUTE_QUERY_PROP`             The query to get the attributes for each instance |
| `static java.lang.String` | `INSTANCE_CLASS_QUERY_PROP`             The query to get the class value for each instance |
| `static java.lang.String` | `NUMBER_OF_ATTRIBUTES`             the total number of possible attributes for the dataset |
| `static java.lang.String` | `NUMBER_OF_CLASS_VALUES`             the total number of possible class values for the dataset |
| `static int` | `NUMBER_OF_CORE_PROPERTIES`             the number of properties that should be available on the properties file (check) |
| `static java.lang.String` | `NUMBER_OF_INSTANCES`             the number of instances (rows) in the arff file |
| `static int` | `NUMBER_OF_STATISTICS_PROPERTIES`             the number of properties + number of data statistics (check: should be 9 + 3) |
| `static java.lang.String` | `TIMESTAMP_FIELD`             the timestamp field |

**Fields inherited from class uk.ac.ed.inf.utils.Initialised**

`m_initialisationIsCorrect`


**Constructor Summary**

`ArffProperties(java.lang.String propertiesFilePath)`
  
          Constructor loads the properties from file


| **Method Summary** | |
| --- | --- |
| `boolean` | `checkPropertiesKeys(java.util.Properties props)` |
| `java.util.TreeSet<java.lang.String>` | `coreKeys()`             The core ARFF properties, essential to generate a Weka ARFF file |
| `boolean` | `corePropertiesAreCorrect(java.util.Properties props)` |
| `java.lang.String` | `getArffFilePath()`             Get the path to the ARFF file |
| `uk.ac.ed.inf.utils.database.DbConn` | `getDbConn()` |
| `java.util.Properties` | `getProperties()`             Get the properties |
| `java.lang.String` | `getPropertiesFilePath()`             Get the properties file path |
| `java.lang.String` | `getProperty(java.lang.String propertyName)`             Get a property value |
| `boolean` | `propertiesAreCorrect(java.util.Properties props)` |
| `java.util.TreeSet<java.lang.String>` | `propertiesKeys()`             All properties: core + statistics |
| `void` | `saveStatisticsAsProperties(java.lang.String numberOfAttributes, java.lang.String numberOfClassValues, java.lang.String numberOfInstances)` |
| `java.util.TreeSet<java.lang.String>` | `statisticsKeys()`             The data statistics properties (number of attributes, classes, instances) added after the arff file generation |
| `boolean` | `statsPropertiesAreCorrect(java.util.Properties props)` |
| `java.lang.String` | `toString()` |

**Methods inherited from class uk.ac.ed.inf.utils.Initialised**

`initialisationIsCorrect, setInitialisationIsCorrect`

**Methods inherited from class java.lang.Object**

`equals, getClass, hashCode, notify, notifyAll, wait, wait, wait`

**Field Detail**

### ARFF\_FILE\_PATH\_PROP

```
public static final java.lang.String ARFF_FILE_PATH_PROP
```

:   the file path where the ARFF file will be written, inclusive of file
    name. A timestamp and the .arff ending will be added automatically.

    **See Also:**: Constant Field Values

---


### CLASS\_NAME\_PROP

```
public static final java.lang.String CLASS_NAME_PROP
```

:   The class name. Examples, for a medical prediction: has\_disease.

    **See Also:**: Constant Field Values

---


### DATA\_PROPERTIES\_FILE\_PATH\_PROP

```
public static final java.lang.String DATA_PROPERTIES_FILE_PATH_PROP
```

:   the path to the properties file itself, from which the other properties
    were read

    **See Also:**: Constant Field Values

---


### DATASET\_NAME\_PROP

```
public static final java.lang.String DATASET_NAME_PROP
```

:   the experiment short name

    **See Also:**: Constant Field Values

---


### DESCRIPTION\_PROP

```
public static final java.lang.String DESCRIPTION_PROP
```

**See Also:**: Constant Field Values

---


### INSTANCE\_ATTRIBUTE\_QUERY\_PROP

```
public static final java.lang.String INSTANCE_ATTRIBUTE_QUERY_PROP
```

:   The query to get the attributes for each instance

    **See Also:**: Constant Field Values

---


### INSTANCE\_CLASS\_QUERY\_PROP

```
public static final java.lang.String INSTANCE_CLASS_QUERY_PROP
```

:   The query to get the class value for each instance

    **See Also:**: Constant Field Values

---


### NUMBER\_OF\_ATTRIBUTES

```
public static final java.lang.String NUMBER_OF_ATTRIBUTES
```

:   the total number of possible attributes for the dataset

    **See Also:**: Constant Field Values

---


### NUMBER\_OF\_CLASS\_VALUES

```
public static final java.lang.String NUMBER_OF_CLASS_VALUES
```

:   the total number of possible class values for the dataset

    **See Also:**: Constant Field Values

---


### NUMBER\_OF\_INSTANCES

```
public static final java.lang.String NUMBER_OF_INSTANCES
```

:   the number of instances (rows) in the arff file

    **See Also:**: Constant Field Values

---


### NUMBER\_OF\_CORE\_PROPERTIES

```
public static final int NUMBER_OF_CORE_PROPERTIES
```

:   the number of properties that should be available on the properties file
    (check)

    **See Also:**: Constant Field Values

---


### NUMBER\_OF\_STATISTICS\_PROPERTIES

```
public static final int NUMBER_OF_STATISTICS_PROPERTIES
```

:   the number of properties + number of data statistics (check: should be 9
    + 3)

    **See Also:**: Constant Field Values

---


### TIMESTAMP\_FIELD

```
public static final java.lang.String TIMESTAMP_FIELD
```

:   the timestamp field

    **See Also:**: Constant Field Values


**Constructor Detail**

### ArffProperties

```
public ArffProperties(java.lang.String propertiesFilePath)
```

:   Constructor loads the properties from file

    **Parameters:**: `propertiesFilePath` - the path to the properties file e.g. /home/me/myexperiment.props


**Method Detail**

### checkPropertiesKeys

```
public boolean checkPropertiesKeys(java.util.Properties props)
```

---


### coreKeys

```
public java.util.TreeSet<java.lang.String> coreKeys()
```

:   The core ARFF properties, essential to generate a Weka ARFF file

    :   **Returns:**: the essential properties to generate an arff file

---


### corePropertiesAreCorrect

```
public boolean corePropertiesAreCorrect(java.util.Properties props)
```

---


### getArffFilePath

```
public java.lang.String getArffFilePath()
```

:   Get the path to the ARFF file

    :   **Returns:**: the path to the ARFF file

---


### getDbConn

```
public uk.ac.ed.inf.utils.database.DbConn getDbConn()
```

---


### getProperties

```
public java.util.Properties getProperties()
```

:   Get the properties

---


### getPropertiesFilePath

```
public java.lang.String getPropertiesFilePath()
```

:   Get the properties file path

---


### getProperty

```
public java.lang.String getProperty(java.lang.String propertyName)
```

:   Get a property value

    :   **Parameters:**: `propertyName` - the name of the property (key) **Returns:**: the associated property value

---


### propertiesAreCorrect

```
public boolean propertiesAreCorrect(java.util.Properties props)
```

---


### propertiesKeys

```
public java.util.TreeSet<java.lang.String> propertiesKeys()
```

:   All properties: core + statistics

    :   **Returns:**

---


### saveStatisticsAsProperties

```
public void saveStatisticsAsProperties(java.lang.String numberOfAttributes,
                                       java.lang.String numberOfClassValues,
                                       java.lang.String numberOfInstances)
```

---


### statisticsKeys

```
public java.util.TreeSet<java.lang.String> statisticsKeys()
```

:   The data statistics properties (number of attributes, classes, instances)
    added after the arff file generation

    :   **Returns:**

---


### statsPropertiesAreCorrect

```
public boolean statsPropertiesAreCorrect(java.util.Properties props)
```

---


### toString

```
public java.lang.String toString()
```

:   **Overrides:**: `toString` in class `java.lang.Object`


---


|  |  |  |
| --- | --- | --- |
| **Overview** \| **Package** \| **Class** \| **Use** \| **Tree** \| **Deprecated** \| **Index** \| **Help** | |  |
| **PREV CLASS**   **NEXT CLASS** | **FRAMES**    **NO FRAMES**     **All Classes** |  |
| SUMMARY: NESTED \| FIELD \| CONSTR \| METHOD | DETAIL: FIELD \| CONSTR \| METHOD |  |


---
